# Correlating the Densities of Hunter-schreger Bands With Function and Surfaces of Teeth: A Micrometric Analysis

**DOI:** 10.12688/f1000research.175647.2

**Published:** 2026-04-18

**Authors:** Radhika Agarwal, Lavangi Sehgal, Jefferson Prince, Srikant Natarajan, Shreya Bhat, Siambiakthang Valte

**Affiliations:** 1 Post graduate student,Department of Orthodontics and Dentofacial Orthopedics, Manipal College of Dental Sciences Mangalore, Manipal Academy of Higher Education, Manipal, Karnataka, 576104, India; 2 Post graduate student ,Department of Prosthodontics and Crown and Bridge, Manav Rachna Dental College, Faridabad, Haryana, 121010, India; 3 Associate Professor,Department of Oral and Maxillofacial Surgery, Manipal College of Dental Sciences Mangalore, Manipal Academy of Higher Education, Manipal, Karnataka, 576 104, India; 4 Professor, Department of Oral Pathology and Microbiology, Manipal College of Dental Sciences Mangalore, Manipal Academy of Higher Education, Manipal, Karnataka, 576104, India; 5 Post graduate student ,Department of Paediatric and Preventive Dentistry, Manipal College of Dental Sciences, Manipal Academy of Higher Education, Manipal, Karnataka, 576 104, India; 6 Post graduate student, Department of Conservative Dentistry and Endodontics, KLE Society's Institute of Dental Sciences, Belagavi, Karnataka, 590010, India

**Keywords:** Hunter–Schreger bands, enamel microstructure, tooth surface, micrometric analysis

## Abstract

**Background:**

Hunter-Schreger bands (HSBs) are distinctive optical features observed in enamel and are characterized by alternating dark and light bands that reflect the orientation of the enamel prism. Despite their clinical relevance, variations in HSB patterns across different tooth surfaces remain poorly understood. *The aim of this study* wass to correlate the width and density of HSB with the surface (labial, lingual, mesial distal and occlusal) of the tooth and the type of tooth.

**Materials and Methods:**

Eighty intact permanent human teeth (20 each: incisors, canines, premolars, molars) from the maxillary and mandibular arches were sectioned in mesiodistal or labiolingual planes. Reflected-light photomicrographs (4× objective; Olympus CX20i) were analysed with ImageJ. HSB density was defined as the number of HSB pairs per millimetre (one dark + one light = pair). Group differences were assessed via repeated-measures ANOVA and independent t tests; *p*< 0.05.

**Statistical analyses:**

Repeated-measures ANOVA and independent t tests were used to assess differences of HSB densities among surfaces and tooth types. A p value of of *p*<0.05 was considered .

**Results:**

Significant variations in HSB density were observed across tooth surfaces within each tooth type and between the maxillary and mandibular arches. Compared with molars, mandibular buccal surfaces presented greater mean HSB densities in premolars ( *p*=0.001) ( *p*=0.201),whereas lingual surfaces presented consistent patterns across all tooth types ( *p*<0.05). Notably, incisal/occlusal surfaces demonstrated the greatest variability, with mandibular incisors exhibiting greater densities ( *p*<0.001) than maxillary incisors ( *p*<0.001).

**Conclusion:**

This micrometric analysis provides detailed insights into the microstructural variations in the HSB across tooth surfaces, highlighting functional and anatomical influences. These findings underscore the clinical importance of HSB in adhesive restorations and enamel-related conditions, suggesting tailored approaches on the basis of tooth type and surface location. Further research is needed to explore the biomechanical implications and refine the clinical applications in dental practice.

**Abbreviations**
ANOVA: analysis of variance
HSB: Hunter–Schreger band
SD: standard deviation
SPSS: Statistical Package for the Social Sciences

## 1. Introduction

Hunter-Schreger bands (HSBs) are optical phenomena in which alternating dark and light bands appear when light is reflected off an enamel surface ^.^. ^1^ Microscopic studies reveal a complex arrangement of enamel rods, which group into bundles or bands with different orientations relative to the tooth’s longitudinal section. Some of these rod groups appear longitudinally sectioned, whereas others seem to be cut transversely or obliquely. ^2^ These longitudinally sectioned groups are referred to as parazones, and those sectioned transversely are known as diazones. The interplay between parazones and diazones has been the foundation for Hunter-Schreger bands in enamel. Some researchers attribute these bands to differences in the density of calcification between alternating zones, whereas others suggest variations in the organic content between the enamel zones. ^3, 4^ Recent synchrotron imaging of developing molars has demonstrated that enamel prisms generally follow a straight path but exhibit irregular, wave-like deviations in three-dimensional space. ^5^ HSB in different teeth of human dentition differ in terms of pattern and packing density. These properties of the HSB have been poorly investigated thus far, and the literature offers little explanation for their distribution. ^6^

The clinical significance of this study is to the differences and how they hold significant relevance for contemporary dental practice, particularly in procedures such as adhesive restoration bonding and in understanding enamel-related conditions such as cracked tooth syndrome and abfraction. ^1^

The aim of this study was to correlate the width and density of HSB with the surface (labial, lingual, mesial distal and occlusal) of the tooth and the type of tooth. The objectives are to measure the thickness of HSB in cross sections of teeth via reflected light microscopy; to correlate the density of HSB with occlusal, labial, lingual, mesial, and distal aspects of teeth; and to list the patterns of arrangement of Hunter-Schreger bands on human teeth on the basis of masticatory and occlusal forces (type traits of the tooth).

## 2. Methodology

### 2.1 Sample calculation

This study was approved by the Institutional Ethics Committee (IEC ref. no. 23094). The extracted human teeth were collected from the Department of Oral and Maxillofacial Surgery; all the samples were anonymized and used following institutional guidelines. Tooth samples that had intact crowns; no evidence of attrition, abrasion, or erosion; and were visually free from hypoplasia and fractures were included in the study. On the basis of the study by Lynch et al. (2010), ^1^ Table 1 shows the HSB densities on various tooth types. The highest standard deviation was noted in premolars, at 2.2 units. Sample size was determined from published variability in HSB density ^1^ where the largest observed standard deviation was approximately σ = 2.2. For a two-sample comparison, using a two-sided α = 0.05 and power = 0.80, the required sample size per group to detect a between-group difference of d = 4.0 units is approximately n ≈ 5 per group (calculation based on the standard two-sample formula). Given the study design (incisor/canine/premolar/molar × maxilla/mandible with two section orientations), we allocated 80 teeth in total (10 teeth per tooth class per arch, further subdivided into two sectioning orientations of n = 5 each). Because some subgroup cells are small, subgroup analyses and interaction effects should be interpreted cautiously; we therefore provide effect sizes and 95% confidence intervals alongside p-values.

**Table 1.** Independent t tests to compare the densities of HSB between the maxilla and mandible.

Tooth	Aspect	Maxilla (n=5) Mean ± SD	Mandible (n=5) Mean ± SD	t	P value
Incisor	Buccal	0.91±0.32	1.04±0.15	-0.814	0.439
	Lingual	1.05±0.29	0.96±0.16	0.589	0.572
	Mesial	0.75±0.17	0.99±0.23	-1.786	0.112
	Distal	0.83±0.22	0.98±0.12	-1.37	0.208
	Incisal/occlusal	0.51±0.12	1.17±0.14	-7.944	**<0.001**
Canine	Buccal	1.06±0.17	1.04±0.18	0.151	0.884
	Lingual	1.01±0.12	0.92±0.18	0.956	0.367
	Mesial	1.03±0.13	0.96±0.18	0.779	0.458
	Distal	1.04±0.07	0.96±0.12	1.31	0.227
	Incisal/occlusal	0.91±0.11	0.7±0.16	2.416	**0.042**
Premolar	Buccal	1.06±0.1	1.5±0.16	5.276	**0.001**
	Lingual	1.02±0.15	1.42±0.22	3.342	**0.01**
	Mesial	0.98±0.12	1.24±0.24	2.134	0.065
	Distal	0.94±0.11	1.11±0.13	2.307	0.05
	Incisal/occlusal	0.86±0.11	1.03±0.15	2.079	0.071
Molar	Buccal	1.29±0.11	1.42±0.19	1.395	0.201
	Lingual	1.44±0.14	1.57±0.13	1.605	0.147
	Mesial	1.42±0.11	1.29±0.12	1.864	0.099
	Distal	1.5±0.24	1.32±0.08	1.561	0.157
	Incisal/occlusal	1.34±0.11	1.37±0.18	0.32	0.757

The formula used is $N=\frac{2{\left(Z_{1-\frac{\alpha }{2*k}}+Z_{1-\beta }\right)}^{2}\sigma^{2}}{d^{2}}$ where σ is the standard deviation.

**Figure 1.**
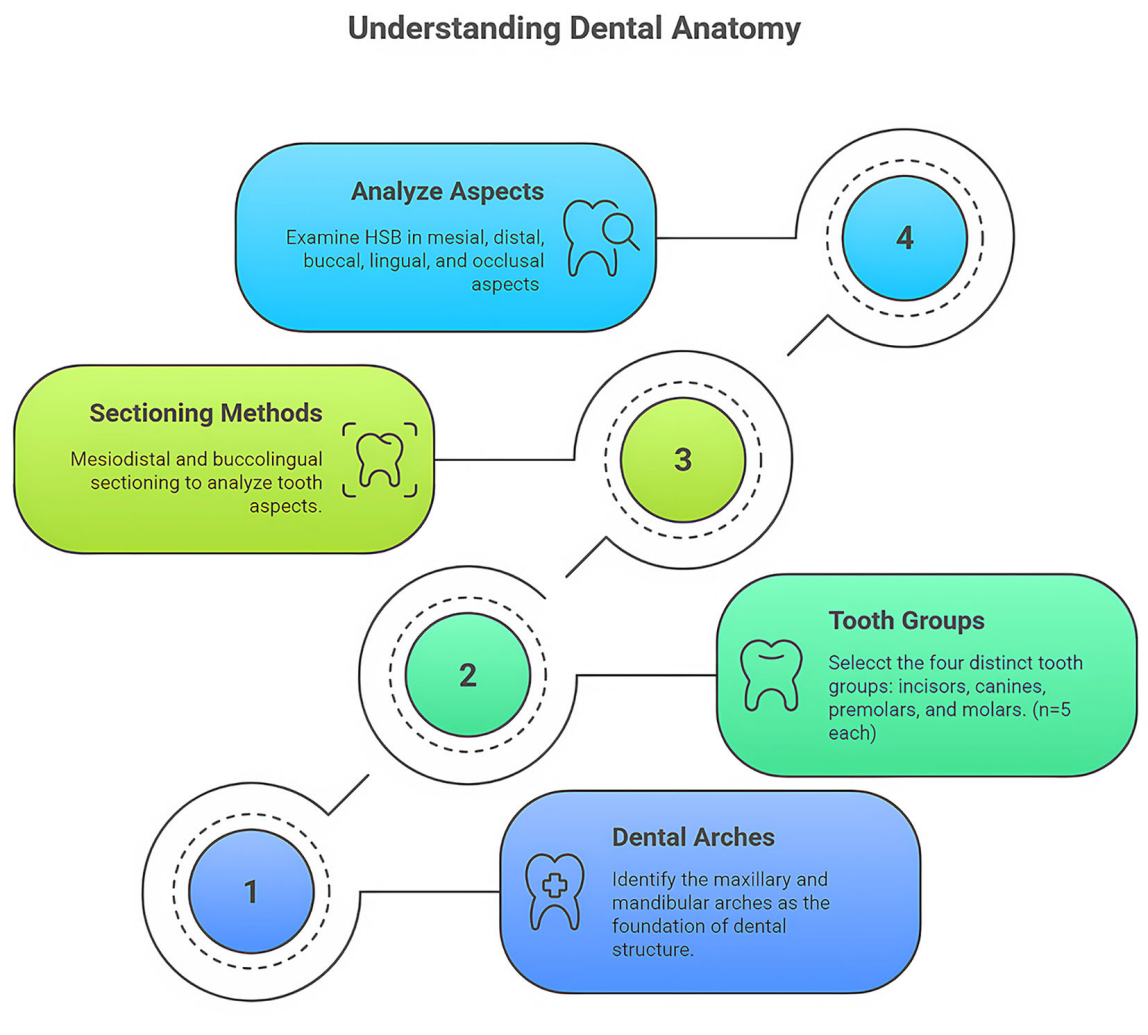
Tooth-class allocation and sectioning methods used in the study: Maxillary and mandibular arches.

Eighty teeth were selected and segregated into incisors, canines, premolars, and molars. A total of 10 samples were included in each category for both jaws, maxillary and mandible. Furthermore, each category was subdivided into 2 groups of five each. One group was used for mesiodistal sectioning, and the other was used for labiolingual sectioning. The occlusal aspect was also studied in each of the above groups. The distribution of teeth is shown in Figure 1.

### 2.2 Preparation of samples

A total of eighty wax molds, each measuring 2 × 2 inches, were fabricated. A thin mix of dental stone was poured into each mold, and once the initial set had occurred, the tooth was positioned such that half of the crown and root were embedded while the remaining portion projected outward. The samples were divided into two groups for mesiodistal and labiolingual sectioning, as both planes are essential for evaluating specific anatomical and microstructural aspects of tooth morphology, particularly the orientation and distribution of Hunter–Schreger bands (HSBs).

Each specimen was first sectioned along the mesio-distal plane, which enabled simultaneous visualization of the mesial, distal, and occlusal surfaces. This orientation facilitated the assessment of band patterns in relation to proximal surfaces and their continuity toward the occlusal aspect.

Subsequently, the same specimens were sectioned in the buccolingual plane to allow visualization of the buccal, lingual, and occlusal surfaces. This approach ensured comprehensive assessment of enamel banding patterns in both orthogonal planes, thereby enabling evaluation of structural variations across the entire crown.

**Figure 2.**
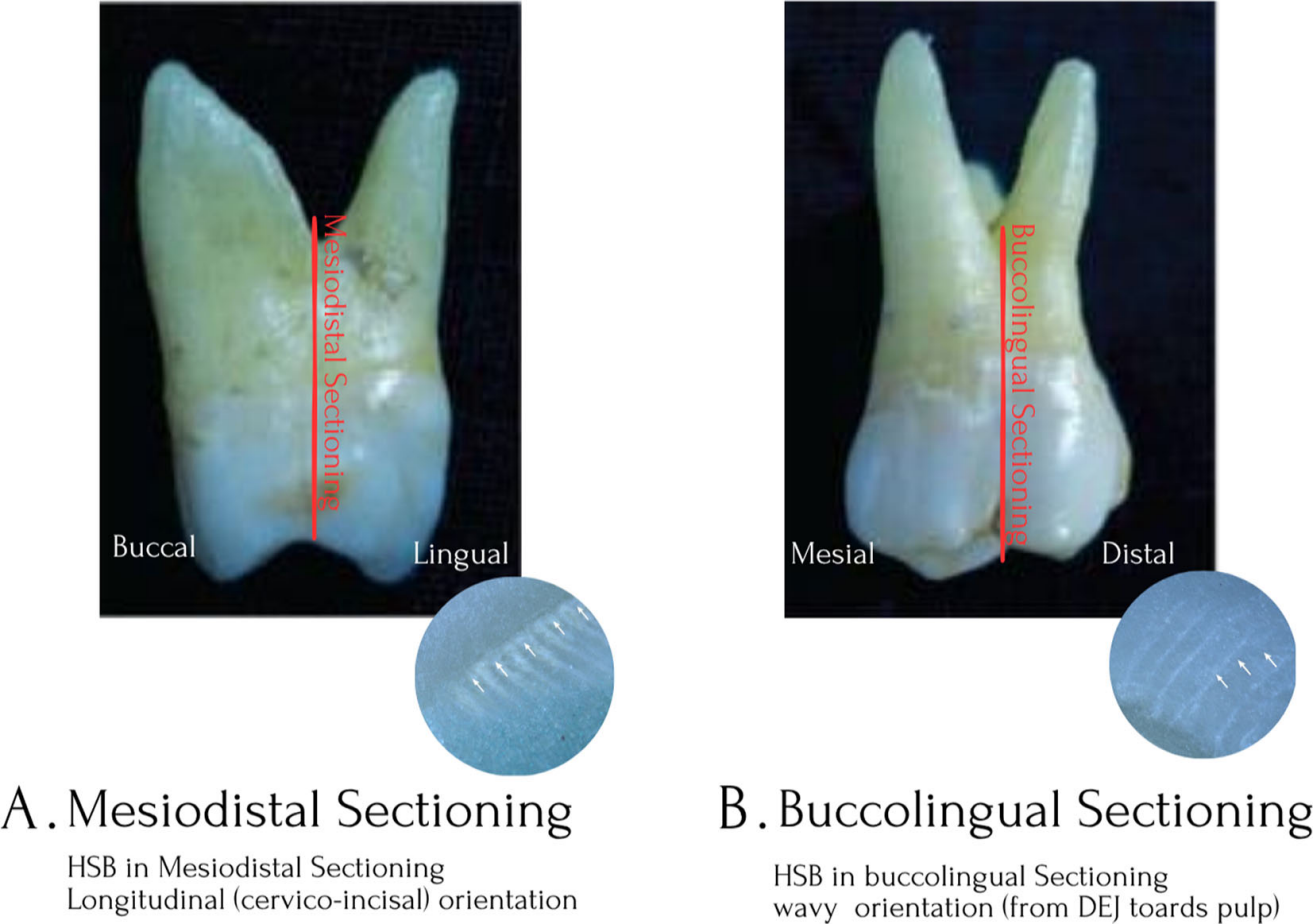
Comparative representation of mesiodistal and buccolingual sectioning of a tooth with corresponding photomicrographs of Hunter–Schreger bands. (A) Mesiodistal section showing crown width and longitudinal orientation of Hunter–Schreger bands (Scale bar = 100 μm). (B) Buccolingual section illustrating crown thickness and comparatively parallel pattern of Hunter–Schreger bands (Scale bar = 100 μm).

The rationale for employing both mesio-distal and buccolingual sectioning was based on the variation in functional loading and stress distribution across different tooth surfaces. In maxillary teeth, the palatal cusps function as the primary load-bearing cusps, whereas in mandibular teeth, the buccal cusps serve this role. Additionally, mesial and distal surfaces are subjected to differing stress patterns during mastication. These functional differences were considered critical for interpreting the orientation and distribution of Hunter–Schreger bands in relation to biomechanical forces ( Figure 2).

Two sectioning orientations were thus followed: one aligning the mesiodistal plane with the study plane and the other aligning the labiolingual plane. After complete setting, the specimens were mounted on a lathe, and half of the exposed tooth surface was carefully reduced to achieve a sectioned half-profile, similar to the methodology described by Lynch et al., ^1^ who emphasized controlled reduction to preserve structural detail. To improve visualization, the samples were subsequently polished using ground sectioning followed by pumice paste. Finally, the cleaned and prepared specimens were subjected to imaging.

### 2.3 Imaging

**Figure 3.**
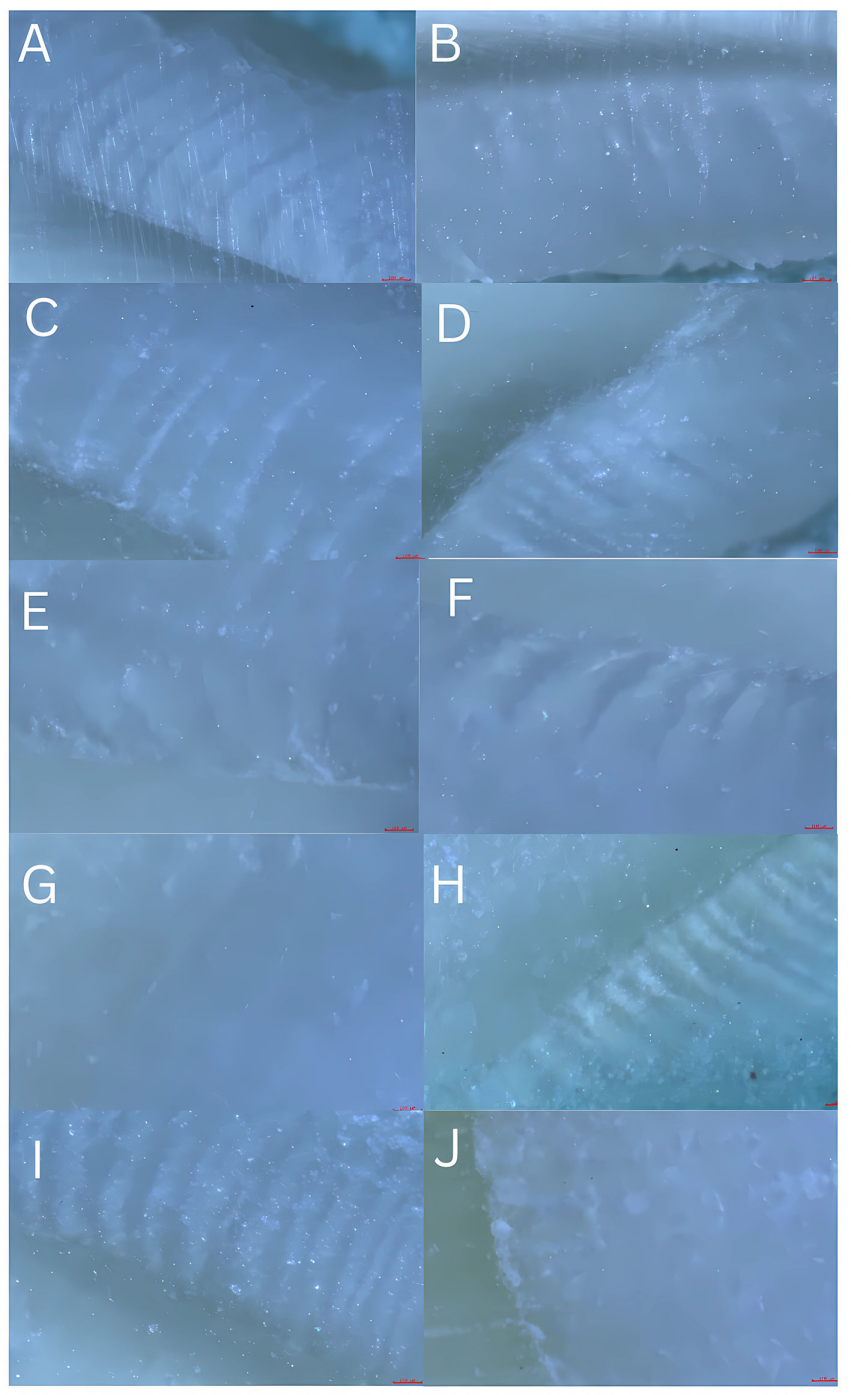
Representative reflected-light photomicrographs showing HSBs on different tooth surfaces. Panels: (A) maxillary incisor labial; (B) maxillary incisor lingual; (C) maxillary canine labial; (D) maxillary canine lingual; (E) mandibular premolar buccal; (F) mandibular molar buccal; (G) mandibular molar lingual; (H) mandibular molar occlusal; (I) mandibular molar lingual; (J) the mandibular molar occlusal. Images captured with 4× objective (Olympus CX20i); scale bar = 100 μm. Original TIFFs archived at Zenodo DOI: 10.5281/zenodo.17919950. Original unprocessed TIFF photomicrographs are available in the Zenodo deposit (see Data Availability) — file names correspond to the samples reported in Table 1.

Photomicrographs were captured using the microscope camera (Olympus CX20i) at a 4× objective and exported as uncompressed TIFF files. Images were calibrated with a stage micrometer and analysed in ImageJ version 1.59 (published by the National Institutes of Health, NIH, USA) as depicted in Figure 3. The number of HSB pairs was counted within defined ROIs (1 dark + 1 adjacent light band = 1 pair).

The density of the HSB was calculated as follows:

$$\left[\text{Density}=\frac{\text{Number of bands}}{\text{Total length of enamel in which the bands}\;\mathrm{are}\;\text{present}\;}\right]$$

The density of the HSB was then tabulated in Microsoft Excel and coded for the surface or the type of tooth.

### 2.4 Reliability

A subset of images (n = 10) was re-analysed by the primary observer and independently analysed by a second observer. Intraclass correlation coefficients (ICC) were calculated using a two-way mixed-effects model for absolute agreement (single measures). 

### 2.5 Statistical analysis

Statistical analyses were conducted using SPSS version 20.0 (IBM Corp., Armonk, NY). Normality of continuous variables was assessed with the Shapiro–Wilk test. Between-arch comparisons used independent-samples t-tests (two-sided) with Cohen’s d reported as the effect size (Hedges’ g reported when n is small). Within-tooth surface differences were assessed with repeated-measures ANOVA (surface as the within-subject factor; arch and tooth class as between-subject factors). Mauchly’s test was used to assess sphericity; where violated, Greenhouse–Geisser or Huynh–Feldt corrections are reported. Partial eta-squared (ηp ^2^) is reported as the effect size for ANOVA. Post hoc pairwise comparisons used Bonferroni correction. All tests used α = 0.05. Exact p-values, degrees of freedom, F statistics, effect sizes and 95% CIs are reported in text and tables.

## 3. Results

### 3.1 Independent t tests to compare the densities of HSB

The most pronounced differences in HSB density were observed on the incisal/occlusal surfaces, with mandibular incisors showing greater densities than their maxillary counterparts did (1.17 ± 0.14 vs. 0.51 ± 0.12, *p* < 0.001) (see Table 1). This finding suggests a stronger adaptive response to masticatory forces in mandibular incisors. Similarly, a substantial difference was noted in the incisal/occlusal surface of the canines, where maxillary canines presented higher HSB density than mandibular canines did (0.91 ± 0.11 vs. 0.70 ± 0.16, *p* = 0.042), possibly reflecting differences in functional demands. Premolars consistently demonstrated greater HSB densities in mandibular teeth, especially on the buccal and lingual surfaces, with a notable difference on the buccal side (1.50 ± 0.16 vs. 1.06 ± 0.10, *p* = 0.001) ( Table 1). In contrast, molars presented minimal variation, with no statistically significant differences across most surfaces, indicating a more uniform HSB distribution in this tooth class.

### 3.2 Measurement reliability

Intra-rater reliability was good (ICC = 0.87, 95% CI 0.74–0.94), indicating consistent measurement of HSB density across repeated trials. Inter-rater reliability was good (ICC = 0.84, 95% CI 0.69–0.93), demonstrating strong agreement between the two observers.

### 3.3 Repeated-measures ANOVA comparing HSB density

Repeated-measures ANOVA was used to assess the impact of the dental surface (Buccal, Lingual, Mesial, Distal, and Incisal/Occlusal) on Hunter-Schreger Band (HSB) density across different tooth classes (Incisor, Canine, Premolar, Molar) in both the maxilla and mandible. The analysis revealed several key findings: ( Table 2).

**Table 2.** Repeated-measures ANOVA comparing HSB density across tooth surfaces, arches, and tooth types.

Source		Type III sum of squares	df	Mean square	F	P value	ηp ^2^
Surface (Side)	Huynh-Feldt	0.947	4.000	0.237	9.617	**<0.001**	0.231032
Surface x Arch	Huynh-Feldt	0.172	4.000	0.043	1.744	0.144	0.051745
Surface x Tooth Type	Huynh-Feldt	0.525	12.000	0.044	1.776	0.059	0.142779
Surface x Arch x Type	Huynh-Feldt	1.009	12.000	0.084	3.413	**<0.001**	0.24249
Error (side)	Huynh-Feldt	3.152	128.000	0.025			

The main effect of arch (Surface × Arch) was not statistically significant (F(4,128) = 1.744, p = 0.144, partial ηp ^2^ = 0.052), indicating no consistent global arch difference when averaged across tooth types and surfaces. However, specific tooth×surface comparisons ( Table 1) revealed localized differences (for example, mandibular incisors and mandibular premolar buccal/lingual surfaces), indicating localized rather than global arch effects.

### 3.4 Effect of surface morphology/texture

The analysis revealed noteworthy differences in the HSB density across the five surfaces (F = 9.617, *p* < 0.001; Table 2). This suggests that the HSB density is not uniformly distributed across all the tooth surfaces. Specifically, the incisal/occlusal surfaces generally presented lower densities than did the other surfaces. For example, the incisal/occlusal surface of mandibular incisors was denser than that of maxillary incisors (mean ± SD: 1.17 ± 0.14 vs. 0.51 ± 0.12, *p* < 0.001; Table 2). Similarly, mandibular premolars presented greater HSB density on the buccal and lingual surfaces than did maxillary premolars (buccal: 1.50 ± 0.16 vs. 1.06 ± 0.10, p = 0.001; lingual: 1.42 ± 0.22 vs. 1.02 ± 0.15, *p* = 0.010; Table 2).

### 3.5 Effect of arch form

No noteworthy differences were observed between the maxilla and mandible regarding the surfaces of the teeth (F = 1.744, *p* = 0.144; Table 2). This indicates that, overall, the distribution of the HSB density is relatively consistent between the two arches when it is averaged across all tooth classes and surfaces. For example, while mandibular premolars and molars tended to show greater densities on the buccal and lingual surfaces, these differences were not statistically significant across all tooth classes (e.g., buccal:maxilla 1.06 ± 0.10 vs. mandible 1.50 ± 0.16, *p* = 0.001).

### 3.6 Interaction between tooth class and surface

The interaction effect between tooth class and surface type was nearly significant (F = 1.776, *p* = 0.059). This suggests that variations in HSB density across different surfaces may differ depending on the tooth class. For example, premolars showed more pronounced differences in HSB density between the buccal and lingual surfaces than incisors did (buccal: 1.06 ± 0.10 for premolars vs. 0.91 ± 0.32 for incisors; lingual: 1.42 ± 0.22 for premolars vs. 1.05 ± 0.29 for incisors; Table 2), indicating that premolars exhibit more variation in response to functional forces.

### 3.7 Three-way interaction among the tooth class, arch, and surface

A significant three-way interaction was found among tooth class, arch, and surface (F = 3.413, *p* < 0.001; Table 2). This major interaction highlights that the effect of the tooth class and surface on the HSB density is influenced by the arch in which the teeth are located. For example:

Mandibular molars had similar HSB densities on the buccal and lingual surfaces to those of maxillary molars (buccal: 1.29 ± 0.11 vs. 1.42 ± 0.19; lingual: 1.44 ± 0.14 vs. 1.57 ± 0.13), but differences were observed in other surfaces, such as the incisor/Occlusal (mandibular molars: 1.37 ± 0.18 vs. maxillary molars: 1.34 ± 0.11, *p* = 0.757; Table 2).

## 4. Discussion

Hunter–Schreger bands (HSBs) are alternating light and dark bands of enamel created by prism decussation. In addition to optical phenomena, they play a biomechanical role by enhancing the resistance of enamel to crack propagation and stress concentration during mastication. ^1^ The present study demonstrated variations in HSB density across tooth types, surfaces, and arches, reflecting the adaptive response of enamel to functional demands.

The observed variations may also be explained by enamel development. Ameloblast displacement during enamel formation alters prism orientation, resulting in denser decussation in regions designed to resist greater mechanical forces. ^3^ In contrast, areas such as cusp tips and cervical enamel may present a reduced HSB density, possibly due to overcrowded ameloblasts or a reduced nutrient supply, resulting in aprismatic enamel or prisms lacking decussation. ^8^

Clinically, these variations in HSB density are highly relevant. Regions of **high HSB density** (mandibular incisors, mandibular premolars, and maxillary canines) exhibit superior resistance to crack propagation and may provide more reliable bonding substrates for adhesive restorations. Conversely, regions of low HSB density (maxillary incisal edges, cervical enamel, some cusp tips) are prone to enamel wear, abfraction, and crack initiation, as reported in studies on cracked tooth syndrome and enamel fracture behaviour. ^9– 12^ These findings align with reports linking cracked tooth syndrome further corroborate that enamel regions with reduced microstructural reinforcement, particularly those with sparse HSB decussation, are more vulnerable to crack propagation under functional loading. ^9^

Compared with maxillary incisors, mandibular incisors presented significantly greater HSB densities on the incisal surface ( *p* < 0.001). This correlates well with its function as the lower incisors have the role of cutting and are directly subjected to shear and compressive forces during incising. A deeper understanding of these teeth functions as a crack-stopping mechanism, reducing fracture risk in thin enamel zones. ^1, 3^ A recent meta-analysis confirmed that the HSB density is greater in functionally stressed enamel regions, particularly the middle enamel third, than in the cervical third. ^7^ In contrast, maxillary incisors act largely as guiding teeth, facing lower direct occlusal loads, hence their reduced density.

Compared with mandibular canines, maxillary canines presented greater HSB densities at the cusp tip/incisal edge ( *p* = 0.042). Maxillary canines are central to **canine guidance**, dissipating large lateral and protrusive forces during mandibular excursions. Denser HSB decussation in this region enhances crack resistance under multidirectional stresses. ^5, 7^ Mandibular canines, although important for aesthetics and occlusal stability, bear comparatively less excursive load, explaining the lower density observed.

Mandibular premolars consistently presented higher HSB densities than did maxillary premolars, particularly on the buccal and lingual surfaces ( *p* = 0.001 and *p* = 0.010). Functionally, mandibular premolars play a dual role in shearing and grinding, especially in unilateral mastication cycles, which subject them to oblique functional stresses. ^11^ Comparative micromechanical assessments indicate that premolars tend to display higher enamel hardness and elastic modulus values compared to canines and incisors. This observation highlights the structural adaptation of premolar enamel to withstand greater functional demands within this tooth class.

In contrast, molars presented relatively uniform HSB densities across arches and surfaces, with no substantial differences. This is consistent with their role as **primary grinding units**, where forces are widely distributed across cusps and thick enamel layers. ^1^ Evolutionary studies indicate that molars adopt consistent HSB configurations to maximize wear resistance, with enamel prisms frequently oriented perpendicularly to occlusal surfaces for optimal reinforcement. ^12, 13^ Thus, unlike incisors and premolars, molars benefit more from uniform reinforcement across surfaces.

Repeated-measures ANOVA confirmed noteworthy differences across surfaces ( *p* < 0.001). The functional surfaces (incisal, buccal, and lingual) presented greater HSB density than did the mesial and distal surfaces. This observation reflects enamel’s adaptive reinforcement of **load-bearing surfaces**. ^1, 3^ The greatest variability was observed at incisal/occlusal surfaces, likely due to differences in stress distribution: mandibular incisors and premolars withstand higher shear loads, whereas maxillary incisors act more as guiding teeth.

HSB packing and decussation patterns likely reflect long-term functional demands on enamel shaped by diet and masticatory behaviour across populations. While the present results are consistent with an adaptive response of enamel microstructure to mechanical loading, testing evolutionary hypotheses requires population-level comparative data and higher-resolution 3-D imaging—topics for future work. ^7, 14, 15^

This study was limited by its modest sample size and reliance on two-dimensional sectioning, which may not fully represent the three-dimensional complexity of the HSB architecture. Future research using high-resolution 3D imaging and Nano indentation testing may provide a deeper understanding of how HSB orientation correlates with enamel strength. Furthermore, exploring HSB adaptations in attached or older teeth across diverse populations could reveal age-related changes in enamel biomechanics.

## 5. Conclusion

This study revealed significant differences in Hunter-Schreger Band density across various tooth types and surfaces, reflecting adaptations to their functional roles. Compared with maxillary incisors, mandibular incisors exhibit notably greater HSB densities on the incisal/occlusal surfaces, suggesting an adaptation to greater masticatory forces. Maxillary canines present higher HSB densities than mandibular canines do, potentially indicating different functional demands. Additionally, mandibular premolars consistently display higher HSB densities on the buccal and lingual surfaces than their maxillary counterparts do, whereas molars show relatively uniform HSB densities across surfaces. These variations in HSB density highlight the specialized adaptation of dental tissues to their biomechanical environments.

## Ethics approval statement

This study was done in collaboration between Manipal College of Dental Science, Mangalore, Manipal Academy of Higher Education, Manipal, India. This study was approved by the Institutional Ethics Committee of Manipal College of Dental Science, Mangalore (IEC Reference number: 23094). The requirement for written informed consent was waived. All research was performed in accordance with the Declaration of Helsinki.

## Patient consent

Written informed consent was obtained from all participants for the use of extracted teeth. The requirement for written informed consent was waived. All research was performed in accordance with the Declaration of Helsinki.

## Preregistered data analysis

The study was not preregistered. No formal data analysis plan was registered in an independent registry prior to data collection or analysis.

## Declaration of generative AI and AI-assisted technologies in the manuscript preparation process

During the preparation of this work the author(s) used ChatGPT (OpenAI) in order to assist with language refinement, clarity improvement, and editing suggestions. After using this tool, the author(s) carefully reviewed and edited the content as needed and take full responsibility for the content of the publication.

## Data Availability

**Zenodo.** *Correlating the densities of Hunter–Schreger bands with function and surfaces of teeth: underlying data.* https://doi.org/10.5281/zenodo.17919950. ^16^ **Notes on image files.** The zip file in the Zenodo deposit contains the original, unprocessed reflected-light photomicrographs used to generate the measurements in this study. The images are provided to ensure full transparency and reproducibility of image cropping, ROI placement and measurement steps. This project contains the following underlying data:
•
**values.xlsx** (Raw HSB counts, ROI lengths, and calculated HSB density values for all samples). • Uncompressed TIFF photomicrographs used for quantitative analysis and representative figures). • TABLE 1 and 2 excel files for the statistical results. Data are available under the terms of the Creative Commons Attribution 4.0 International (CC BY 4.0) license.
