# Supplementary material for: Correlating the Densities of Hunter-schreger Bands With Function and Surfaces of Teeth: A Micrometric Analysis
Source: F1000Res. 2026 Apr 18;15:354. Originally published 2026 Mar 4. [Version 2] doi: 10.12688/f1000research.175647.2 (PMC13070207; doi:10.12688/f1000research.175647.2)
Supplement: Peer review history

# Reviewer report: Reviewer response for version 1

**DOI:** 10.5256/f1000research.193650.r471054

**Published:** 2026-04-11

**Authors:** Riaz Abdulla

**Affiliations:** 1 Yenepoya Dental College, Mangalore, India

The manuscript is well written and presents a scientifically sound study with an appropriate statistical analysis. The topic is relevant, and the findings contribute valuable insights into the distribution and functional significance of the Hunter–Schreger bands. The data availability and transparency were commendable. However, minor clarifications in the methodology, particularly regarding the occlusal surface evaluation and justification of the sectioning techniques, are necessary to ensure full reproducibility. Addressing these issues will further strengthen the manuscript.

Is the work clearly and accurately presented and does it cite the current literature?

Yes

If applicable, is the statistical analysis and its interpretation appropriate?

Yes

Are all the source data underlying the results available to ensure full reproducibility?

Yes

Is the study design appropriate and is the work technically sound?

Yes

Are the conclusions drawn adequately supported by the results?

Yes

Are sufficient details of methods and analysis provided to allow replication by others?

Partly

Reviewer Expertise:

oral pathology,oral cancer

I confirm that I have read this submission and believe that I have an appropriate level of expertise to confirm that it is of an acceptable scientific standard.

# Reviewer report: Reviewer response for version 1

**DOI:** 10.5256/f1000research.193650.r469099

**Published:** 2026-03-25

**Authors:** L S Makesh Raj

**Affiliations:** 1 TN Dr. MGR Medical University, Tagore Dental College and Hospital, Chennai, Tamil Nadu, India

The article is well written and presents a strong statistical analysis. However, a few points in the Methodology section require clarification to ensure reproducibility:

1. **Criteria for evaluating the occlusal aspect**
  The manuscript mentions that “the occlusal aspect was also studied in each of the above groups,” but the specific criteria or parameters used for this evaluation are not described. Please clarify what features were assessed or standards followed.
2. **Justification for sectioning approach**
  The rationale behind dividing the samples into two groups—one for mesiodistal sectioning and the other for labiolingual sectioning—is not clearly explained. Kindly provide the reasoning for this methodological choice and how it contributes to the study objectives or improves the analysis.

Providing these details will enhance the clarity and reproducibility of the study.

Is the work clearly and accurately presented and does it cite the current literature?

Yes

If applicable, is the statistical analysis and its interpretation appropriate?

Yes

Are all the source data underlying the results available to ensure full reproducibility?

Yes

Is the study design appropriate and is the work technically sound?

Yes

Are the conclusions drawn adequately supported by the results?

Yes

Are sufficient details of methods and analysis provided to allow replication by others?

Partly

Reviewer Expertise:

oral Pathology and Forensic Odontology

I confirm that I have read this submission and believe that I have an appropriate level of expertise to confirm that it is of an acceptable scientific standard.

# Response

**Published:** 2026-04-01

**Authors:** Srikant Natarajan

**Affiliations:** Manipal College of Dental Sciences Mangalore, India

1. We thank the reviewer for highlighting this point. The occlusal aspect was evaluated to assess the appearance and orientation of Hunter–Schreger bands at the cusp tips and occlusal enamel surface. Specifically, the following features were considered: (1) visibility and clarity of banding patterns, (2) orientation of bands relative to the cusp slopes, and (3) continuity or disruption of bands from the subsurface enamel toward the occlusal surface. This assessment was performed qualitatively under magnification to correlate surface expression with underlying band patterns observed in sectional planes. The corresponding details have now been added to the revised manuscript. (Figure 2)

 2. We appreciate the reviewer’s observation. The division of samples into mesiodistal and buccolingual sectioning groups was undertaken to enable comprehensive evaluation of enamel banding patterns in two orthogonal planes. Mesiodistal sectioning allowed visualization of mesial, distal, and occlusal surfaces; similarly, buccolingual sectioning enabled evaluation of buccal, lingual, and occlusal aspects, providing complementary information on band orientation across the crown as mentioned in Figure 2.

 This approach was essential because enamel microstructure, including Hunter–Schreger bands, varies with functional loading and stress distribution. Maxillary palatal cusps and mandibular buccal cusps serve as primary load-bearing areas, while proximal surfaces experience different stress patterns during mastication. Assessing specimens in both planes allowed for a more accurate interpretation of band orientation and distribution in relation to these biomechanical factors, thereby strengthening the study’s analytical framework.
